# Genotyping of circulating tumor DNA in cholangiocarcinoma reveals diagnostic and prognostic information

**DOI:** 10.1038/s41598-019-49860-0

**Published:** 2019-09-13

**Authors:** T. J. Ettrich, D. Schwerdel, A. Dolnik, F. Beuter, T. J. Blätte, S. A. Schmidt, N. Stanescu-Siegmund, J. Steinacker, R. Marienfeld, A. Kleger, L. Bullinger, T. Seufferlein, A. W. Berger

**Affiliations:** 1 University Medical Center Ulm, Center for Internal Medicine, Department of Internal Medicine I, University of Ulm, Ulm, Germany; 2 Charité University Medical Center Berlin, Department of Hematology, Oncology and Tumorimmunology, Berlin, Germany; 3 University Medical Center Ulm, Center for Internal Medicine, Department of Internal Medicine III, University of Ulm, Ulm, Germany; 4 University Medical Center Ulm, Department of Diagnostic and Interventional Radiology, University of Ulm, Ulm, Germany; 5 University Medical Center Ulm, Institute of Pathology, University of Ulm, Ulm, Germany; 6 Department of Gastroenterology, Gastrointestinal Oncology and Interventional Endoscopy, Vivantes Klinikum im Friedrichshain, Teaching Hospital of Charité - University Medical Center Berlin, Berlin, Germany

**Keywords:** Tumour heterogeneity, Translational research, Cancer genetics, Bile duct cancer

## Abstract

Diagnosis of Cholangiocarcinoma (CCA) is difficult, thus a noninvasive approach towards (i) assessing and (ii) monitoring the tumor-specific mutational profile is desirable to improve diagnosis and tailor treatment. Tumor tissue and corresponding ctDNA samples were collected from patients with CCA prior to and during chemotherapy and were subjected to deep sequencing of 15 genes frequently mutated in CCA. A set of ctDNA samples was also submitted for 710 gene oncopanel sequencing to identify progression signatures. The blood/tissue concordance was 74% overall and 92% for intrahepatic tumors only. Variant allele frequency (VAF) in ctDNA correlated with tumor load and in the group of intrahepatic CCA with PFS. 63% of therapy naive patients had their mutational profile changed during chemotherapy. A set of 76 potential progression driver genes was identified among 710 candidates. The molecular landscape of CCA is accessible via ctDNA. This could be helpful to facilitate diagnosis and personalize and adapt therapeutic strategies.

## Introduction

Cholangiocarcinoma including intrahepatic cholangiocarcinoma (IHCC), extrahepatic cholangiocarcinoma (EHCC) and gallbladder cancer, are rare types of cancer with an incidence of 0.45 to 3.5/100000 in the Western world^1–3^. Only a minority of patients are diagnosed at a resectable disease stage and relapse rates after curative surgery are high. Cisplatin/gemcitabine combination chemotherapy has shown efficacy as a 1^st^ line palliative treatment regimen and 1-year survival rate has slightly improved^4^. Currently there is no standard treatment regimen in further therapy lines and no targeted therapies are established^5^. New therapeutic options are currently under evaluation, in 1^st^ line^6^ or following therapy lines [ClinicalTrials.gov Identifier: NCT03044587]^6,7^. Diagnosis is normally based on imaging and tumor tissue analysis, however often hampered. Tissue biopsies are sometimes difficult to obtain, in particular in case of extrahepatic and hilar localization of the cholangiocarcinoma (CCA). Moreover, sensitivity of brush cytology via endoscopic retrograde cholangiopancreatography (ERCP), transpapillary tumor biopsy or endoscopic ultrasound-guided fine needle aspiration (EUS-FNA) for histological diagnosis is low^8^, leading to repeated biopsies and delayed diagnosis. Molecular characterization is not yet recommended by international guidelines^5,9^. Although the risk factors for IHCC or EHCC partially overlap they are biologically distinct diseases. Hepatolithiasis, chronic hepatitis B/C, diabetes and obesity are risk factors for IHCC, while chronic cholangitis for EHCC^10^. Mutations that impair DNA mismatch repair like Lynch syndrome are a strong risk factor for both tumor types (IHCC and EHCC)^11^. TP53 and KRAS mutations were significantly associated with a worse survival and are more frequent in EHCC, while mutations and fusions in FGFR1-3, IDH1/2 and ARID1A were more frequent in IHCC^12^. These specific differences in the mutational landscape open for separate therapeutic targeting for example with the dual BCR/ABL and Src family tyrosine kinase inhibitor dasatinib (NCT02428855) or AG120 and IDH1-inhibitors (NCT02073994). Moreover, the commonly found chronic inflammation in CCAs makes them a potentially good candidate for immunotherapeutic approaches as shown with first promising results in phase 2 trial^13^. There is increasing evidence that systemic chemotherapy drives a Darwinian type of tumor evolution^14^. To monitor this evolution, repeated tissue biopsies could be performed but are cumbersome for the patient and may also be of limited use due to intratumoral heterogeneity^15^.

A promising approach towards a simple access to the tumor’s molecular profile is the use of circulating tumor DNA (ctDNA). However, data on ctDNA from CCA patients are sparse. Pancreatic, biliary and liver cancer patients are frequently pooled in literature, which makes the study results unclear. These tumors are different in carcinogenesis, incidence and clinical management and should be viewed as separate entities. Due to these substantial differences we indeed need facile, preferably non-invasive, tools to differentiate between HCC and CCA in patients with liver lesions, for example. Precision medicine could also be substantially supported by tools that allow monitoring of the tumor’s molecular profile over the time course of chemotherapy. These gaps could be filled by the analysis of ctDNA.

The major aim of our study was to evaluate whether ctDNA targeted genotyping is suitable for (i) non-invasive assessment of the tumor-specific mutational profile and (ii) the monitoring of this profile in locally advanced and metastatic CCA undergoing 1^st^ line palliative chemotherapy.

## Results

### Mutational landscape of therapy naive cholangiocarcinoma

**Figure 1:**
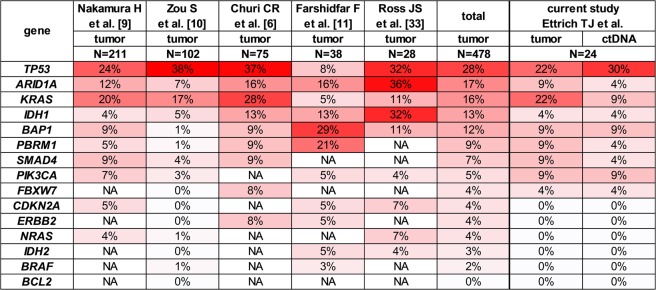
Comparison of mutational frequencies across 15 driver cancer genes in tumors and ctDNA obtained in this study shows high degree of correlation with published datasets of cholangiocarcinoma. Genes are sorted by mean mutation frequency. NA - not available.

13/24 tumors (54%) were categorized as intrahepatic cholangiocarcinoma (IHCC), 11 tumors (46%) were extrahepatic cholangiocarcinoma (EHCC). Patient characteristics are shown in Table 1 and specified in more detail in Supplementary Table 1. The respective baseline laboratory findings are shown in detail in Supplementary Table 3. Positive risk factors for cholangiocarcinoma could be could be found in 16/24 (66.7%) patients. The minority of 8/24 (33.3%) patients had no significant risk factors for developing a CCA **(**Supplementary Table 4**)**. 23 therapy naive patients were available for concordance analysis prior to treatment initiation. We did not have access to archived tumor tissue material of 1 patient (#18). ctDNA sampled during 1^st^ line palliative treatment was available from 11 patients and used for tracking of tumor-specific mutations in ctDNA under therapy. ctDNA sampled during 2^nd^ or 3^rd^ line therapy was available from 5 of the 24 patients. Detailed molecular characteristics are provided in Supplementary Table 2. Tumor biopsy material of 23/24 patients was analyzed by targeted enrichment and NGS of 15 genes. We found 22 mutations in 9 of these genes. The mutational frequencies are consistent with the published mutational landscape of CCA (Fig. 1). In our cohort 61% of patients (IHCC: 58%; EHCC: 64%) had at least one mutation in tumor driver genes. On average IHCC harbored 0.83 mutations and EHCC 1.1 mutations per tumor.

**Table 1:** Baseline clinical characteristics of patients in the study.

Characteristic	N (%)
Patients	24
Age (years)	66.7 ± 12.2
Male/Female	15 (63)/9 (38)
Primary tumor location	
Intrahepatic (IHCC)	13 (54)
Extrahepatic (EHCC)	11 (46)
Metastatic sites	
0	3 (13)
IHCC	2 (8)
EHCC	1 (4)
1	11 (46)
IHCC	6 (25)
EHCC	5 (21)
>1	10 (42)
IHCC	5 (21)
EHCC	5 (21)
Tumor stage	
UICC III (all IHCC)	3 (12)
UICC IV	21 (88)
Tumor load at baseline (mm, RECIST 1.1)	
IHCC	107 ± 45
EHCC	69 ± 42
Mean PFS (months)	
IHCC	5.0 ± 2.9
EHCC	4.1 ± 1.9

IHCC - intrahepatic cholangiocarcinoma, EHCC - extrahepatic cholangiocarcinoma, UICC - Union Internationale Contre Le Cancer, PFS - progression-free survival, mm – millimeter.

### Concordance between mutations in tumor tissue and ctDNA in therapy naive patients

**Figure 2:**
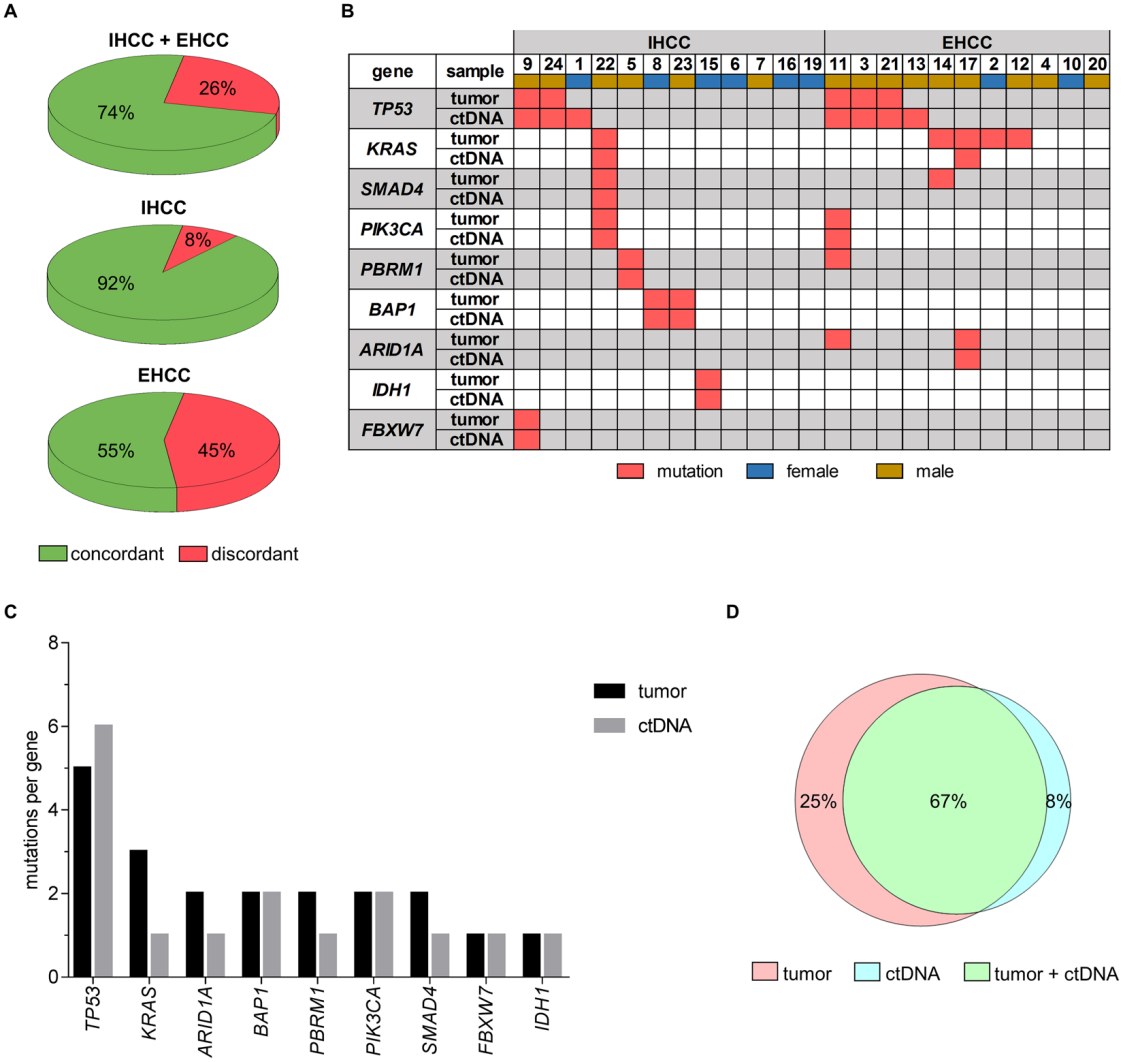
Mutational profile of therapy naive cholangiocarcinoma patients assessed by targeted resequencing analysis of tumor tissue and ctDNA and concordance of mutations detected in tumor tissue and ctDNA of therapy naive cholangiocarcinoma patients. (**A**) Proportion of patients with identical mutational profile in tumor tissue and ctDNA. Data are shown for the entire cohort (top) and grouped by tumor localization, for IHCCs (middle) and for EHCCs only (bottom). (**B**) Detailed mutational profile per patient. Patients were sorted by mutation frequency per gene and separated according to primary tumor localization. Only genes with detected mutations are shown here. (**C**) Number of total unique variants identified per gene. No statistical difference in the mean number of unique variants between tumor and ctDNA (*P* = 0.3125, Wilcoxon) or between IHCC and EHCC patients (tumor p > 0.9999, Wilcoxon; ctDNA *P* = 0.3594, Wilcoxon). (**D**) Venn diagram showing the overlap between ctDNA and tumor biopsy sequencing analysis for every mutation reported. IHCC - intrahepatic cholangiocarcinoma; EHCC - extrahepatic cholangiocarcinoma.

The mutational profile of the 23 available blood-tumor pairs was concordant for 74% of patients. Stratified according to tumor localization, the concordance rate for IHCC was 92%. In contrast, only 55% of EHCC patients had concordant profiles (Fig. 2A). Detected mutations per patient in tissue and ctDNA, respectively, are shown in Fig. 2B.

The number of unique variants that were found per gene was not statistically different between tumor tissue and ctDNA (*P* = 0.3125). The largest number of unique variants was detected in the *TP53* gene (Fig. 2C). Overall, 67% of all mutations were concordant between tumor tissue and ctDNA. 6 tumor mutations (25%) could not be detected in ctDNA, whereas 2 mutations (8%) found in ctDNA were not seen in the respective tumor sample (Fig. 2D).

### Baseline variant allele frequency in tumor tissue and ctDNA

**Figure 3:**
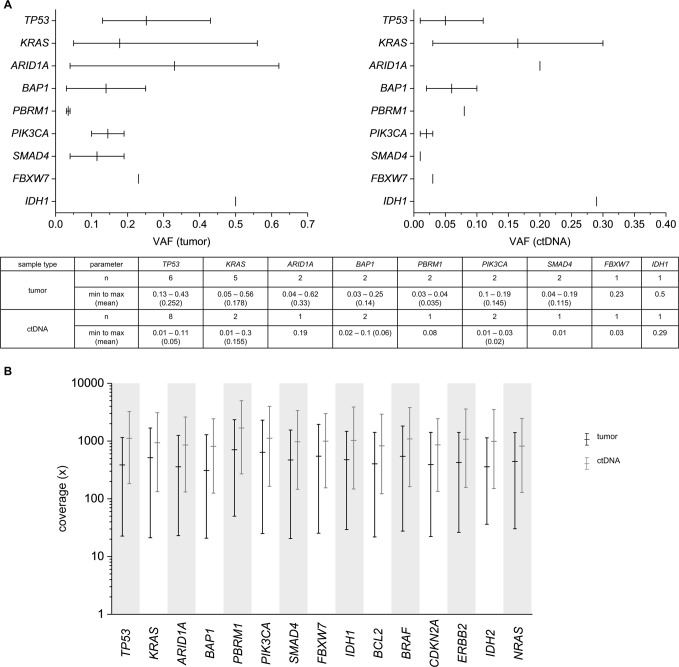
Variant allele frequencies (VAF) and sequencing depth in tumor tissue and ctDNA. (**A**) VAFs of all detected variants per gene in tumor tissue (left) and ctDNA (right). Bars indicate range of mutated allele fraction (min, max, mean). (**B**) Coverage statistics per gene across all samples for tumor tissue and ctDNA. Mean total coverage is significantly higher for ctDNA samples (1010x) than for tumor tissue samples (465x; p < 0.0001, Mann-Whitney). Bars indicate range of mutated allele fraction (min, max, mean).

Variant allele frequencies (VAF) in tumor tissue and ctDNA were compared at baseline in therapy naive patients. The observed mean VAF in tumor tissue (0.214) was significantly higher than in ctDNA (0.098) (*P* = 0.0291, Fig. 3A) as expected. Mean sequencing depth across the analyzed genes was significantly higher for ctDNA samples (1010x) than for tumor tissue samples (465x, p < 0.0001) (Fig. 3B).

### Effect of treatment on ctDNA variant allele frequencies

**Figure 4:**
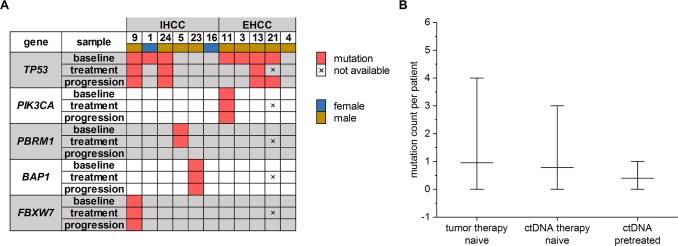
Tumor evolution during treatment based on mutations found in ctDNA. (**A**) Detailed mutational profile of 11 patients receiving 1^st^ line palliative chemotherapy prior to therapy initiation (baseline), 1.7 ± 0.7 months after therapy initiation (treatment) and at radiologically confirmed disease progression (progression). Patients were sorted by mutation frequency per gene and separated according to intrahepatic (IHCC) or extrahepatic (EHCC) primary tumor localization. Only genes with mutations detected are shown here. (**B**) Absolute number of mutations per patient is not significantly different in tumor and ctDNA of therapy naive patients (N = 23, *P* = 0.6997, Mann-Whitney) and between ctDNA samples of therapy naive and pretreated patients (N = 5, *P* = 0.5519, Mann-Whitney). Bars show mean and range of variation.

ctDNA of 11 patients was sequentially analyzed during 1^st^ line palliative chemotherapy. The mutational landscape in ctDNA of 36% of these patients (4/11) changed compared to baseline. Three of them had a *TP53* mutation that was no more detectable after treatment. The fourth patient had a *PBRM1* mutation, which was not seen any more at the “progression” time point. In all cases, sequencing at the respective variant positions was deep enough to ensure the detection of at least 1% variant allele frequency. Mutations emerging during treatment could not be detected in ctDNA within the analyzed genes (Fig. 4A). Moreover, we had access to ctDNA samples of 5 pretreated patients. We compared the number of mutations per patient at baseline in tumor tissue with that in ctDNA of therapy naive patients and with that in ctDNA of pretreated patients: The tumor tissue samples of the therapy naive group (*N* = 23) showed a mean of 0.96 (0–4) mutations. The corresponding ctDNA samples (*N* = 23) showed a mean of 0.78 (0–3) mutations per patient, while ctDNA samples of pretreated patients (*N* = 5) exhibited a mean of 0.4 (0–1) mutations. There were no statistically significant differences in the mutation number per patient between these groups (therapy naive: tumor vs. ctDNA *P* = 0.6997; ctDNA: therapy naive vs. pretreated *P* = 0.5519; Fig. 4B). The selected chemotherapeutic regimen (gemcitabine, gemcitabine/oxaliplatin, gemcitabine/cisplatin) had no influence on VAF changes during treatment (data not shown).

### Prognostic impact of ctDNA variant allele frequencies

ctDNA VAF baseline values in IHCC and EHCC correlated significantly (*P* = 0.0433) with the respective initial tumor load (RECIST 1.1) prior to 1^st^ line palliative treatment (Supplementary Fig. 2A). In both IHCC and EHCC, there was no correlation between the number of detected mutations and the respective tumor load (*P* = 0.9431, data not shown).

Finally, we tested the predictive potential of ctDNA VAF baseline values on progression-free survival (PFS). Among the entire cohort there was a trend but no significant correlation between these parameters (*P* = 0.0530, r = −0.3996, Supplementary Fig. 2B). Looking at IHCC and EHCC independently, there was a significant correlation between baseline ctDNA VAF and PFS in the IHCC group (*P* = 0.0288, r = −0.5878, Supplementary Fig. 2C) which could not be detected in the EHCCs (*P* = 0.2380, r = −0.2974, Supplementary Fig. 3C). The absolute number of detected mutations showed no significant correlation with PFS (*P* = 0.0907, Supplementary Fig. 3D).

### Dynamic levels of CA 19-9 tumor marker

Plasma levels of the disease progression marker carbohydrate antigen 19-9 (CA19-9) were determined in all liquid biopsy samples. The median CA19-9 level decreased from 102.6 IU/ml (1.2–10000 IU/ml) at baseline to 31.2 IU/ml (6.4–2238 IU/ml, *P* = 0.5416) under treatment and increased significantly (*P* = 0.0292) to 157 IU/ml (8.1–2716 IU/ml) at progression time-point. Baseline CA19–9 levels did not correlate with tumor load (*P* = 0.1341, r = −0.3148), variant allele frequency (*P* = 0.7851, r = −0.0550) or PFS (*P* = 0.8614, r = −0.03854).

### Potential progression driver signatures

**Figure 5:**
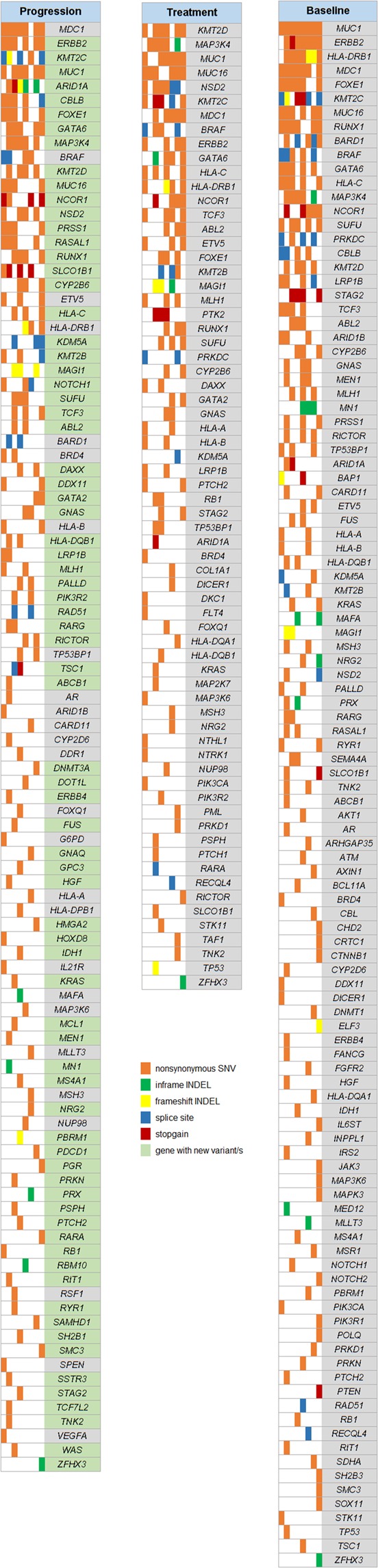
Gene map showing all genes with somatic mutations in exonic/splice regions from 710-gene targeted resequencing of ctDNA samples from 8 individuals with CCA at three time-points: prior to therapy initiation (“Baseline”), under chemotherapy (“Treatment”) and at radiologically confirmed disease progression (“Progression”).

To identify mutation signatures indicating disease progression, ctDNA samples from baseline, treatment and progression time-points of an additional group of 8 patients was submitted to an expanded targeted panel resequencing comprising 710 cancer related genes. We found in total 1442 unique somatic variants, of which 16 were splice variants and 520 were exonic variants. The exonic variants consisted of 301 missense SNVs, 14 in-frame Indels, 10 frameshift indels and 12 stopgain mutations. Figure 5 shows genes that were somatically mutated in exonic or splicing regions at the respective time-point. The total number of mutated genes as well as the average number of mutated samples per gene decreased from baseline (110 genes, 1.6 mutated samples/gene) to treatment time-point (68 genes, 1.0 mutated samples/gene) and increased again from treatment to progression time-point (102 genes, 1.4 mutated samples/gene). Genes in which variants showed up during chemotherapy until disease progression that were not initially detected at baseline are highlighted. The most frequently mutated genes among these were *ERBB2*, *KMT2C* and *MUC1*, which were each mutated in 75% of patients, followed by *ARID1A*, *CBLB*, *FOXE1*, *GATA6* and *MAP3K4* which were each mutated in 62.5% of patients.

## Discussion

The results obtained from NGS based targeted genotyping of tumor tissue from therapy naive CCA patients are in line with previously published data^16–20^. There was a high concordance rate between ctDNA and tissue results of 74% overall and even 92% in IHCC group. The lower concordance (55%) in EHCC may be due to specific features of the anatomic site, but also underlines that IHCC and EHCC may indeed be distinct diseases, with differences in genomic pattern and clinical behavior^21^. This biological variety is mainly driven by the different cell of origin in IHCC and EHCC defining them as independent tumor entities underlined by the divergent mutational spectrum^22–24^. Differences in the mutational spectrum that are demonstrated amongst other things in this paper. Moreover, this triggers the clinical behavior exposing IHCC as the more aggressive disease with accelerated metastasis and worse outcome compared to EHCC^25,26^. This may directly impact the quantity of measurable ctDNA. Nevertheless, tumor heterogeneity can also lead to discordance between tumor tissue DNA and ctDNA profiling^27^. Some mutations detected in ctDNA have prognostic value. Mutations in *BAP1* and *PBRM1*^28^, *KRAS*^16,18^ and *TP53*^16,17^ were previously reported to be associated with poor overall survival, even in the curative disease stage^29^. Our data show a trend towards shorter PFS in patients with a mutation in either of the genes mentioned above compared to patients without any mutation in these genes (Supplementary Fig. 3E). A recent multi-institutional trial on CCA confirmed the correlation of genomic profiles with clinical outcome^30^. Thus, beside diagnostics, the analysis of ctDNA can help to estimate prognosis of patients with CCA. In addition, the mutational status of *CDKN2A*, *TP53* and *ARID1A* was reported to influence treatment response and PFS in CCA^31^. Of note, these reports are based on retrospective analyses of archived tumor tissue material. Prospective, liquid biopsy-based studies are not available so far.

One of our patients (#15) had a potentially actionable mutation in *IDH1* in the baseline blood sample. The biological role of *IDH1* mutations in CCA is currently unclear. While some authors reported a better prognosis with a longer time-to-tumor-recurrence in IHCC^32^, others did not find a prognostic relevance^18,30^. Moreover, Ivosidenib, an inhibitor of mutated *IDH1* has shown encouraging results in a phase I trial including patients with CCA^33^ and is evaluated in a phase III trial in advanced cholangiocarcinoma (ClinicalTrials.gov identifier: NCT02989857). Thus, screening ctDNA for *IDH1* mutations under therapy could indeed help to personalize first line treatment. This also applies for FGFR-altered advanced CCA, where a recently published phase II study (ClinicalTrials.gov identifier: NCT02150967) showed a promising antitumor activity of BGJ398, a selective pan-*FGFR* kinase inhibitor^34^.

Considering the data presented here and the confined number of relevant mutations in CCA previously identified by whole exome sequencing^17^, the targeted approach chosen may be clinically valuable for diagnosis of suspicious findings and estimation of prognosis. The question remained if a broader gene panel could cover the complex mechanism of tumor evolution. To address this question, samples of an additional group of 8 patients were submitted to a large-scale panel sequencing (710 cancer-related genes). The results indicate a certain degree of detectable changes in the molecular constitution during cytotoxic treatment, probably be described as tumor evolution. We hypothesize, that this was most likely triggered by selection pressure through the cytotoxic effects of the chemotherapy employed. We identified a set of 149 out of 710 cancer-related genes that show mutations in CCA before and throughout chemotherapeutic treatment and a subset of 76 genes with variants absent at baseline but emerging under chemotherapy that are probably driving disease progression, suggesting that tumor progress in CCA is a rather heterogenous process and large-scale panels are needed to monitor treatment-induced tumor evolution in this disease. In detail, the most frequently mutated genes at disease progression were *ERBB2*, *KMT2C* and *MUC1*, *ARID1A*, *CBLB*, *FOXE1*, *GATA6* and *MAP3K4*. Beside *ARID1A*, all these genes do not belong to the assumed and published most frequently mutated genes in CCA^16^. *ERBB2* mutations were reported to be druggable^35^. *KMTC2* mutations were not correlated with CCA so far. In diffuse gastric cancer, *KMT2C* is frequently mutated and is associated with worse overall survival^36^. In liver cancer, *MUC1* was described as a prognostic biomarker^37^ and *MAP3K4* deficiency leads to invasive growth and epithelial-mesenchymal transition, namely in IHCC^38^. Reports about *FOXE1* and *CBLB* mutations in CCA are lacking so far but *FOXE1* was described as a new susceptibility locus in thyroid cancer^39^ and CBLB gene mutations were reported to be associated with multi-chemoresistance in breast cancer cell-lines^40^. A few years ago, *GATA6*, a transcriptional regulator previously linked to normal pancreas development, was discussed as a candidate lineage-specific oncogene in pancreaticobiliary cancer, with implications for novel treatment strategies^41^. Conversely, a report from Tian F *et al*. showed that an aberrant expression of *GATA* binding protein 6 correlates with poor prognosis and promotes metastasis in CCA^42^.

However, reports discussed CCA as a genetically diverse cancer^43^ and the precise number of driver genes in CCA is still elusive either in carcinogenesis or in tumor progression. Likewise, we do not have evidence, which event(s) might drive the disease progression in CCA. A number of epigenetic alterations, such as promoter hypermethylation and microRNA dysregulation have been associated with development, biological and clinical behavior of CCA. Epigenome analysis of ctDNA, recently reported as a minimally invasive diagnosis and disease classification tool even of early-stage cancers^44^, has not yet been published in the area of CCA. It is necessary to address these questions in further studies.

In conclusion, ctDNA sequencing harbors great potential to improve the clinical management of CCA patients. Mutations detected in ctDNA are representative for the respective tumor tissue (especially for IHCC), paving the way to a non-invasive molecular diagnosis and therapy stratification. Our data are encouraging for the estimation of the individual tumor load and the expected prognosis, which also might influence treatment decisions. However, it is important to emphasize, that the data analyses followed a descriptive, hypothesis-generating approach resulting in preliminary conclusions. Further investigations on bigger studies are urgently needed.

## Materials and Methods

### Patient characteristics and study design

32 patients with histologically confirmed locally advanced or metastatic CCA (UICC stage III and IV) were enrolled in this study. Gall bladder cancer patients were excluded. Irresectability and indication for palliative chemotherapy were confirmed by the local multidisciplinary tumor conference. Archived FFPE tumor material from initial diagnosis was used for comparison to ctDNA from the time point of initial diagnosis. All ctDNA and tumor tissue DNA samples were submitted to targeted next generation sequencing (NGS). The CONSORT diagram (Supplementary Fig. 1) illustrates patient groups and applied methods.

### Institutional review board

Prior to start of the study a positive vote from the institutional review board of Ulm University was obtained (Ulm University, approval numbers: 317/12, 230/14, 128/15). Participation in the study was voluntary. All patients signed a written informed consent prior to inclusion. All methods were performed in accordance with the relevant guidelines and regulations.

### Sample collection

Blood samples for ctDNA analyses were collected prospectively at predefined time points (“baseline”: prior to treatment initiation; “treatment”: 1.7 ± 0.8 months after treatment initiation; “progression”: at radiologically confirmed disease progression in general 2 weeks beyond last cytotoxic treatment). CT-scans were done at baseline and in intervals of 2.7 ± 1.6 months during treatment; all scans were analyzed according to RECIST 1.1 criteria.

### Plasma collection

7.5 ml of whole venous blood were collected in EDTA tubes (Sarstedt, Nümbrecht, Germany) by peripheral blood draw, kept at 4 °C until separation (within 1 hour after collection). Whole blood was centrifuged for 10 minutes (820 × g at 4 °C), plasma fraction was transferred and subsequently centrifuged again for 10 min (20.000 × g at 4 °C). Pure plasma was recovered in fresh 2 ml tubes for immediate storage at −80 °C until DNA extraction^45^.

### Extraction of ctDNA

Circulating tumor DNA was extracted from plasma using the QIAamp Circulating Nucleic Acid Kit (QIAGEN, Hilden, Germany) according to manufactures instruction and as previously reported^45^. 4 ml of plasma were used for each DNA extraction. Recovered DNA was eluted in 50 μl of elution buffer and stored at −20 °C until further use.

### Isolation of tumor DNA from FFPE tissue

For isolation of tumor DNA from FFPE tissue samples, 5μm tissue slices were transferred to glass slides. To estimate the tumor containing area, hematoxylin and eosin stained FFPE tissue slices (2 µm) were validated by an expert pathologist using conventional light microscopy. The tumor-harboring areas were marked and material was macrodissected and subjected to a DNA extraction procedure using the QIAamp DNA FFPE tissue kit (QIAGEN, Hilden, Germany) according to the manufactures instruction.

### Next generation sequencing

15-gene panel: For molecular characterization of both tumor tissue and ctDNA, we employed a custom targeted enrichment of all exons of the 15 most frequently mutated genes selected based on previously published data of CCA mutational landscape: *TP53, KRAS, ARID1A, BAP1, PBRM1, PIK3CA, SMAD4, FBXW7, IDH1, BCL2*, *BRAF, CDKN2A, ERBB2, IDH2, and NRAS*^16–19,46^. Libraries were generated using custom SureSelectXT enrichment kit (Agilent Technologies, Waldbronn, Germany). Library preparation and sequencing were performed at GATC Biotech (Konstanz, Germany). Paired-end sequencing (2 × 125 bp) was carried out on a HighSeq. 2500 platform (Illumina, San Diego, CA, USA) with a target coverage of 5000x. The applied bioinformatics pipeline followed our previously published workflow^47^. Briefly, after quality control using NGS QC Toolkit_v2.3 the reads were mapped to reference genome hg19 with BWA-MEM 0.7.10 and sequence duplicates were marked with Picard 1.138. GATK (GenomeAnalysis TK-3.4-46) was used for local realignment and variants were called with Varscan v2.3.9. The coverage statistics were assessed using bedtools 2.24.0. Bases with a minimum quality score of 30 were considered for variant calling. Variants presenting with reads in both orientations, with ≥10 supporting reads, a sequencing depth of ≥20 and a frequency of ≥0.01 were reported. The variants were annotated with ANNOVAR release 20150322^48^. Subsequently, polymorphisms annotated in dbSNP (dbSNP147))^49^ and 1000 Genomes (1000G Ph3)^50^ databases were not considered as mutations, except polymorphisms present in human population at frequencies ≤0.001 and reported in COSMIC database^51^. Remaining calls were verified by visual inspection in IGV^52^, low confidence calls were discarded.

710-gene oncopanel: To identify potential signatures for disease progression, an additional cohort of 8 patients was recruited and cfDNA samples from baseline, treatment and progression time-points as well as germline DNA samples isolated from lymphocytes were submitted to a 710-gene targeted resequencing oncopanel by CeGaT GmbH (Tübingen, Germany). Germline variants of each patient were subtracted from the respective cfDNA VCF files using VCFtools version 0.1.13^53^. The resulting variant lists were annotated with ANNOVAR, subsequently filtered for variants in exonic/splice regions, synonymous SNVs were omitted.

### Statistical analyses

Results for continuous variables are presented as mean ± standard deviation (SD) or median ± median absolute deviation (MAD). Groups were compared with (paired/unpaired) Student’s t-test, Mann-Whitney U (unpaired) or Wilcoxon signed rank test (paired). Comparison of categorical variables was generated by Fisher’s exact test. Correlation analyses were performed by Pearson or Spearman correlation analysis. *P*-values < 0.05 were considered significant. Statistical analyses were performed using GraphPad Prism version 7 (GraphPad Software, La Jolla, CA, USA). For illustration OriginPro 2017 (OriginLab Corporation, Northampton, MA, USA) and Venn Diagram Plotter (PNNL, Richland, WA, USA, OMICS.PNL.GOV) were used. Data analyses followed a descriptive, hypothesis-generating approach and therefor has to be termed as preliminary work.

## Supplementary information

Dataset 1

## Data Availability

The datasets generated during and/or analyzed during the current study are available from the corresponding author on reasonable request.
