# Supplementary material for: Genotyping of circulating tumor DNA in cholangiocarcinoma reveals diagnostic and prognostic information
Source: Sci Rep. 2019 Sep 13;9:13261. doi: 10.1038/s41598-019-49860-0 (PMC6744511; doi:10.1038/s41598-019-49860-0)
Supplement: Supplementary file 1 — Dataset 1 [file 41598_2019_49860_MOESM1_ESM.pdf]

Genotyping of circulating tumor DNA in cholangiocarcinoma reveals diagnostic and prognostic information

T. J. Ettrich[1]†, D. Schwerdel[1]†, A. Dolnik[2]†, F. Beuter[1], T. J. Blätte[3], S. A. Schmidt[4], N. Stanescu-Siegmund[4], J. Steinacker[4], R. Marienfeld[5], A. Kleger[1], L. Bullinger[2], T. Seufferlein[1*], A. W. Berger[1,6]

* Corresponding author.

† These authors contributed equally to this work.

[1] University Medical Center Ulm, Center for Internal Medicine, Department of Internal Medicine I, University of Ulm, Ulm, Germany.

[2] Charité University Medical Center Berlin, Department of Hematology, Oncology and Tumorimmunology, Berlin, Germany

[3] University Medical Center Ulm, Center for Internal Medicine, Department of Internal Medicine III, University of Ulm, Ulm, Germany.

[4] University Medical Center Ulm, Department of Diagnostic and Interventional Radiology, University of Ulm, Ulm, Germany

[5] University Medical Center Ulm, Institute of Pathology, University of Ulm, Ulm, Germany

[6] Department of Gastroenterology, Gastrointestinal Oncology and Interventional Endoscopy, Vivantes Klinikum im Friedrichshain, Teaching Hospital of Charité - University Medical Center Berlin, Germany

Corresponding author:

Prof. Dr. med. Thomas Seufferlein

Albert-Einstein-Allee 23

89081 Ulm, Germany

thomas.seufferlein@uniklinik-ulm.de

Supplementary Materials

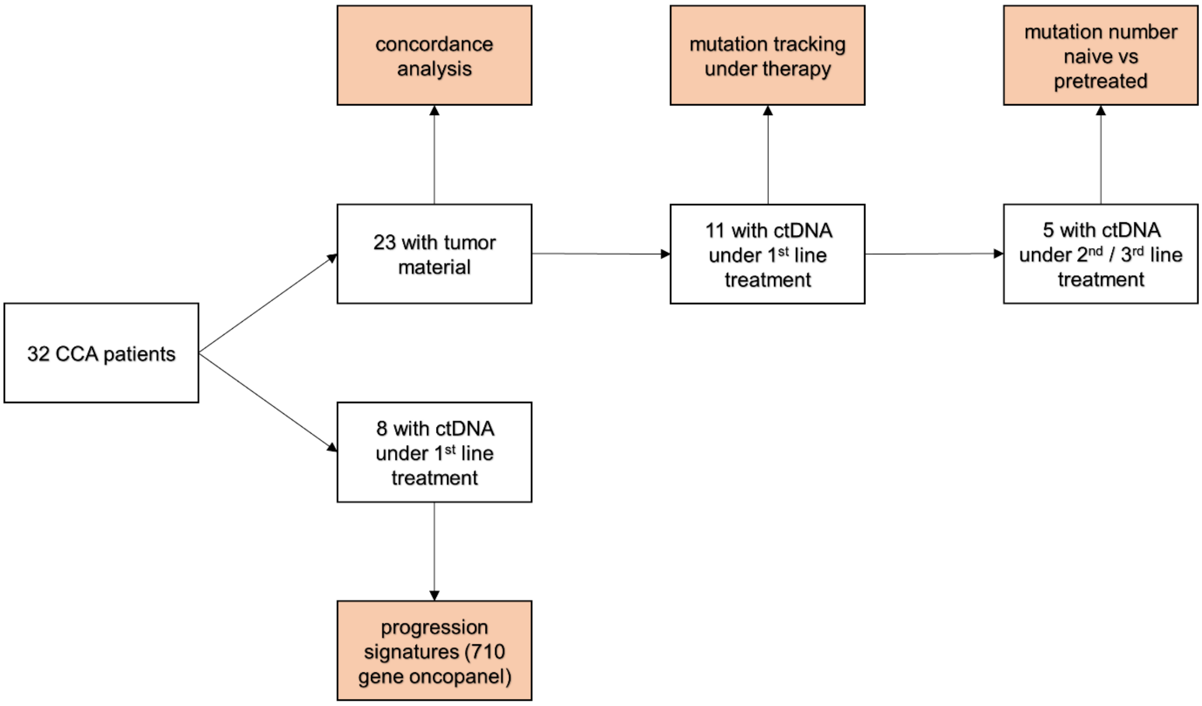

Supplementary Figure 1. Patient groups and applied methods.

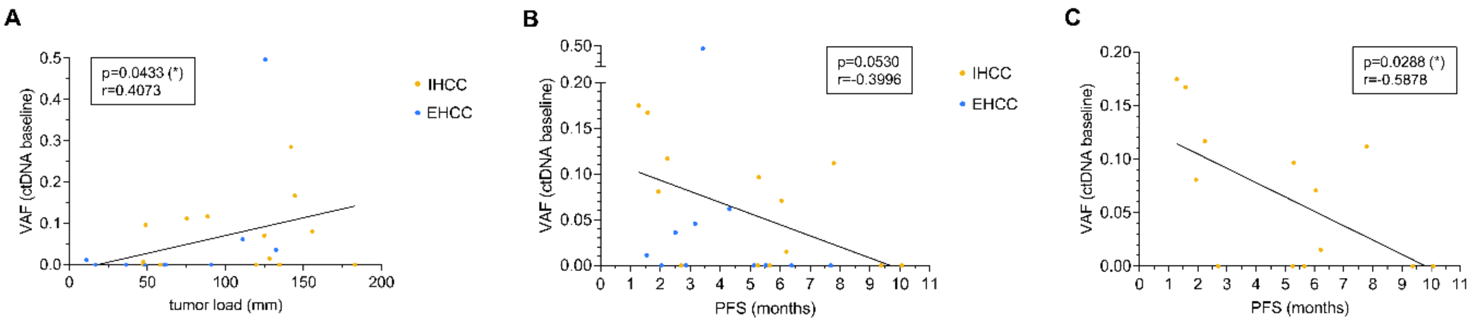

Supplementary Figure 2. Correlation of ctDNA VAF baseline values and clinical parameters. **A.** VAF at baseline correlates with tumor load (RECIST 1.1) at baseline (*P*=0.0433, r=0.4037, Spearman). **B.** Lower VAF at baseline shows a trend towards prolonged progression-free survival (PFS) (*P*=0.0530, r=-0.3996, Spearman). **C.** VAF in the group of IHCC patients correlates with PFS (*P*=0.0288, r=-0.5878, Spearman). IHCC - intrahepatic cholangiocarcinoma, EHCC - extrahepatic cholangiocarcinoma.

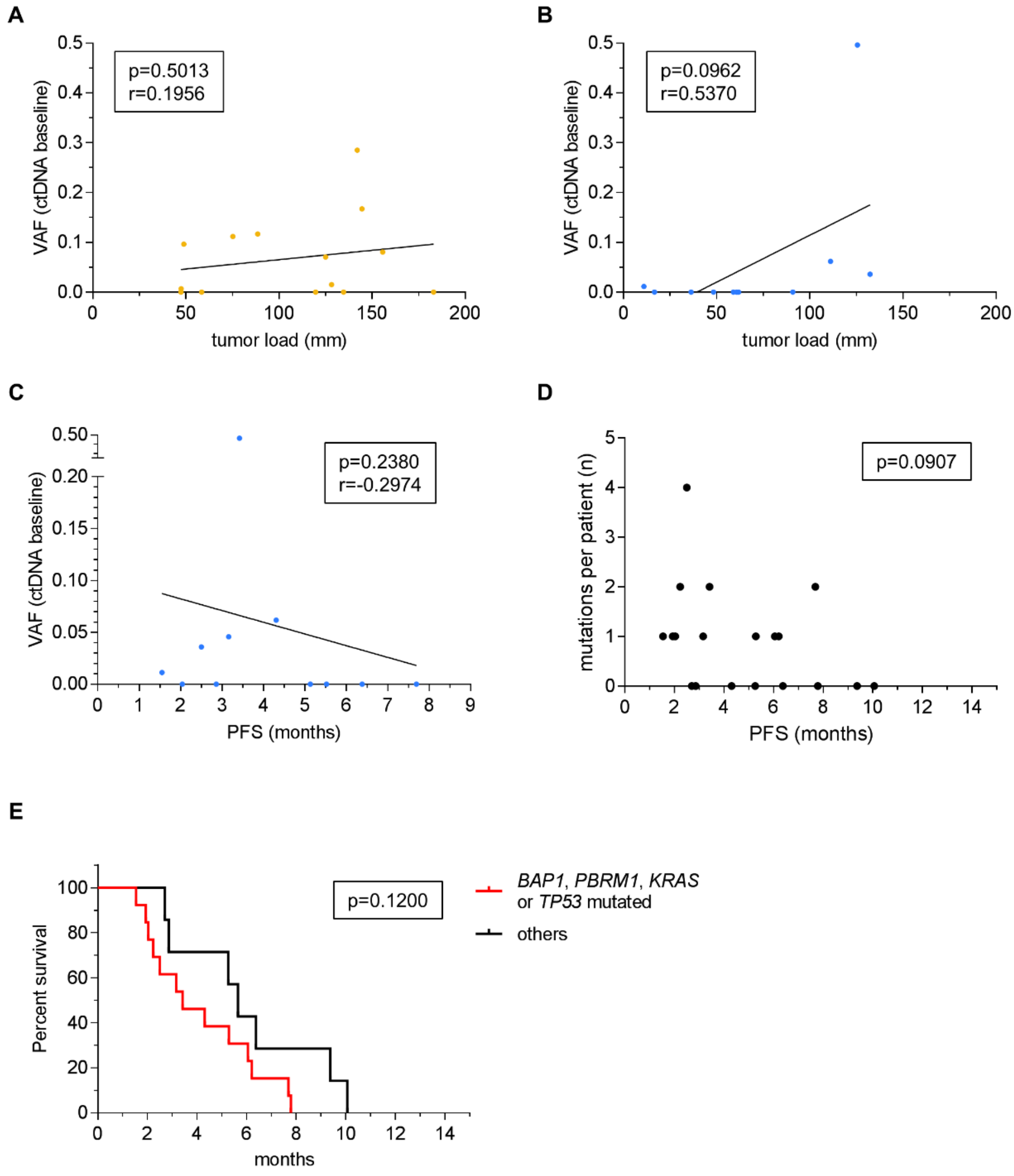

Supplementary Figure 3. Correlation of ctDNA VAF baseline values with **A.** tumor load (RECIST 1.1) in the group of IHCC patients (*P*=0.5013, r=0.1956, Spearman), **B.** tumor load (RECIST 1.1) in the group of EHCC patients (*P*=0.0962, r=0.5370 Spearman), **C.** progression-free survival (PFS) in the group of EHCC patients (*P*=0.2380, r=-0.2974, Spearman). **D.** Correlation of the number of mutations per patient with PFS (*P*=0.0907, r=-0.3988, Spearman). **E.** Kaplan-Meier plot of PFS of patients with mutations in *BAP1*, *PBRM1*, *KRAS* or *TP53* vs. patients without mutations in these genes (*P*=0.1200, Mantel-Cox). IHCC - intrahepatic cholangiocarcinoma, EHCC - extrahepatic cholangiocarcinoma.

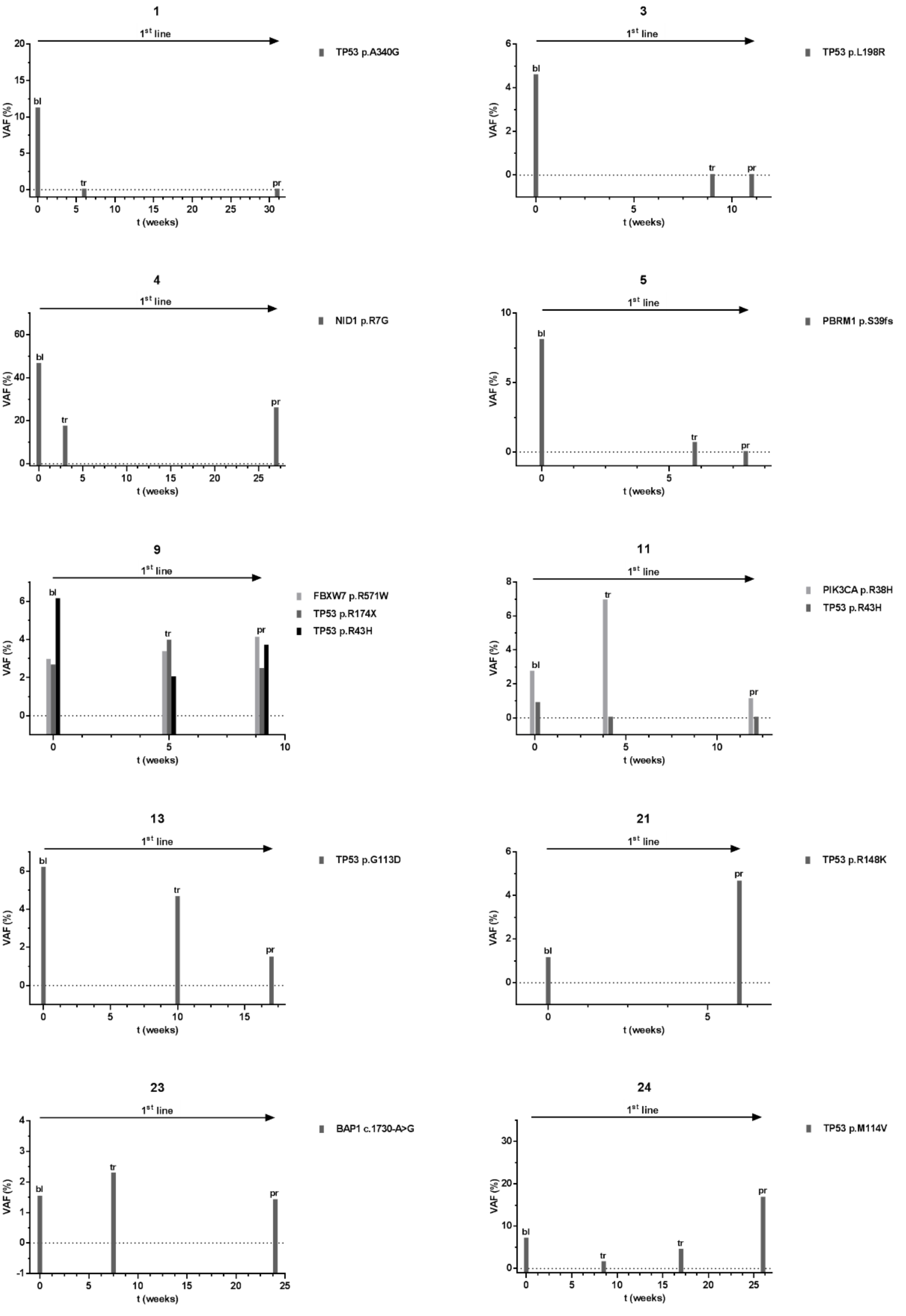

Supplementary Figure 4. Variant allele frequencies of detected mutations of all patients receiving first line chemotherapy from which cfDNA during the course of treatment was available (one patient was wild-type for the applied gene panel). bl - baseline, tr - treatment, pr - progression.

Supplementary Table 1. Clinical patient characteristics.

ID	sex	age	tumor localization	stage	metastases	chemotherapeutic regimen	
						therapy line	chemotherapy
1*	F	59	Intrahepatic	UICC IV	ADR, HEP, LYM, PER, OTH	1st, 2nd	Gem/Cis, FOLFIRI
2	F	78	Extrahepatic	UICC III	-	-	-
3	M	70	Extrahepatic	UICC IV	ADR, PLE	1st	Gem/Cis
4*	M	74	Extrahepatic	UICC IV	HEP, LYM, PUL	1st, 2nd, 3rd	Gem/Cis, FOLFIRI, Doce
5	M	70	Intrahepatic	UICC III	-	1st	Gem
6	F	55	Intrahepatic	UICC IV	ADR, LYM	1st	Gem/Cis
7	M	75	Intrahepatic	UICC III	-	1st	Gem/Cis
8	F	80	Intrahepatic	UICC IV	HEP, OSS, PUL	1st	Gem
9	M	84	Intrahepatic	UICC IV	LYM	1st	Gem
10	F	70	Extrahepatic	UICC IV	PER	1st	Gem/Ox
11	M	62	Extrahepatic	UICC IV	LYM	1st	Gem/Cis
12	M	75	Extrahepatic	UICC IV	PER	1st	Gem/Carbo
13	M	62	Extrahepatic	UICC IV	LYM	1st	Gem/Ox
14	M	74	Extrahepatic	UICC IV	PER	1st	Gem
15	F	54	Intrahepatic	UICC IV	LYM	1st	Gem/Cis
16	F	42	Intrahepatic	UICC IV	HEP, LYM	1st	Gem/Cis
17	M	39	Extrahepatic	UICC IV	LYM, OSS	1st	Gem/Cis
18*	F	59	Intrahepatic	UICC IV	HEP	2nd	FOLFIRI
19	F	49	Intrahepatic	UICC IV	LYM	1st	Gem/Ox
20	M	86	Extrahepatic	UICC IV	LYM, PUL	1st	Gem
21	M	73	Extrahepatic	UICC IV	PER, PUL, HEP, LYM	1st	Gem/Cis
22	M	72	Intrahepatic	UICC IV	LYM	1st	Gem/Ox
23	M	65	Intrahepatic	UICC IV	HEP, OSS, PUL	1st	Gem/Cis
24*	M	59	Intrahepatic	UICC IV	PUL	1st, 2nd	Gem/Cis, FOLFIRI

* = analyzed prior advanced therapy line(s); F = female; M = male; UICC = Union Internationale Contre le Cancer; ADR = adrenal; HEP = hepatic; LYM = lymphoid; OSS = osseous; PER = peritoneal; PUL = pulmonary; OTH = other; Gem = Gemcitabine; Cis = Cisplatin; Doce = Docetaxel; Ox = Oxaliplatin; Carbo = Carboplatin; FOLFIRI = folinic acid/5-fluorouracil/irinotecan

Supplementary Table 2. Molecular characteristics of patients

Tumor biopsy (baseline)

ID	therapy line	TP53 AA-change	TP53 VAF (%)	KRAS AA-change	KRAS VAF (%)	ARID1A AA-change	ARID1A VAF (%)	BAP1 AA-change	BAP1 VAF (%)	PBRM1 AA-change	PBRM1 VAF (%)	PIK3CA AA-change	PIK3CA VAF (%)	SMAD4 AA-change	SMAD4 VAF (%)	FBXW7 AA-change	FBXW7 VAF (%)	IDH1 AA-change	IDH1 VAF (%)	BCL2 AA-change	BCL2 VAF (%)	BRAF AA-change	BRAF VAF (%)	CDKN2A AA-change	CDKN2A VAF (%)	ERBB2 AA-change	ERBB2 VAF (%)	IDH2 AA-change	IDH2 VAF (%)	NRAS AA-change	NRAS VAF (%)
1	1st	-	-	-	-	-	-	-	-	-	-	-	-	-	-	-	-	-	-	-	-	-	-	-	-	-	-	-	-	-	-
2	1st	-	-	p.G12V	5.24	-	-	-	-	-	-	-	-	-	-	-	-	-	-	-	-	-	-	-	-	-	-	-	-	-	-
3	1st	p.L198R	34.78	-	-	-	-	-	-	-	-	-	-	-	-	-	-	-	-	-	-	-	-	-	-	-	-	-	-	-	-
4	1st	-	-	-	-	-	-	-	-	-	-	-	-	-	-	-	-	-	-	-	-	-	-	-	-	-	-	-	-	-	-
5	1st	-	-	-	-	-	-	-	-	p.S39fs	2.7	-	-	-	-	-	-	-	-	-	-	-	-	-	-	-	-	-	-	-	-
6	1st	-	-	-	-	-	-	-	-	-	-	-	-	-	-	-	-	-	-	-	-	-	-	-	-	-	-	-	-	-	-
7	1st	-	-	-	-	-	-	-	-	-	-	-	-	-	-	-	-	-	-	-	-	-	-	-	-	-	-	-	-	-	-
8	1st	-	-	-	-	-	-	p.P124R	25.49	-	-	-	-	-	-	-	-	-	-	-	-	-	-	-	-	-	-	-	-	-	-
9	1st	R174X; p.R43	25.29; 23.57	-	-	-	-	-	-	-	-	-	-	-	-	p.R571W	23.35	-	-	-	-	-	-	-	-	-	-	-	-	-	-
10	1st	-	-	-	-	-	-	-	-	-	-	-	-	-	-	-	-	-	-	-	-	-	-	-	-	-	-	-	-	-	-
11	1st	p.R43H	16.87	-	-	p.D1900G	3.72	-	-	p.S681G	3.55	p.R38H	19.44	-	-	-	-	-	-	-	-	-	-	-	-	-	-	-	-	-	-
12	1st	-	-	p.G12R	6.08	-	-	-	-	-	-	-	-	-	-	-	-	-	-	-	-	-	-	-	-	-	-	-	-	-	-
13	1st	-	-	-	-	-	-	-	-	-	-	-	-	-	-	-	-	-	-	-	-	-	-	-	-	-	-	-	-	-	-
14	1st	-	-	p.G13D	10.51	-	-	-	-	-	-	-	-	p.S325fs	4.21	-	-	-	-	-	-	-	-	-	-	-	-	-	-	-	-
15	1st	-	-	-	-	-	-	-	-	-	-	-	-	-	-	-	-	p.R132C	49.82	-	-	-	-	-	-	-	-	-	-	-	-
16	1st	-	-	-	-	-	-	-	-	-	-	-	-	-	-	-	-	-	-	-	-	-	-	-	-	-	-	-	-	-	-
17	1st	-	-	p.G12V	56.1	p.K974fs	61.76	-	-	-	-	-	-	-	-	-	-	-	-	-	-	-	-	-	-	-	-	-	-	-	-
19	1st	-	-	-	-	-	-	-	-	-	-	-	-	-	-	-	-	-	-	-	-	-	-	-	-	-	-	-	-	-	-
20	1st	-	-	-	-	-	-	-	-	-	-	-	-	-	-	-	-	-	-	-	-	-	-	-	-	-	-	-	-	-	-
21	1st	p.R148K	29.18	-	-	-	-	-	-	-	-	-	-	-	-	-	-	-	-	-	-	-	-	-	-	-	-	-	-	-	-
22	1st	-	-	p.G12V	11.11	-	-	-	-	-	-	p.K111N	9.55	p.R361H	18.82	-	-	-	-	-	-	-	-	-	-	-	-	-	-	-	-
23	1st	-	-	-	-	-	-	c.1730-2A>G	3	-	-	-	-	-	-	-	-	-	-	-	-	-	-	-	-	-	-	-	-	-	-
24	1st	p.M114V	13.33	-	-	-	-	-	-	-	-	-	-	-	-	-	-	-	-	-	-	-	-	-	-	-	-	-	-	-	-

Liquid biopsy (circulating tumor DNA)

ID	therapy line	TP53 AA-change	TP53 VAF (%) baseline	TP53 VAF (%) treatment	TP53 VAF (%) progression	KRAS AA-change	KRAS VAF (%) baseline	KRAS VAF (%) treatment	KRAS VAF (%) progression	ARID1A AA-change	ARID1A VAF (%) baseline	ARID1A VAF (%) treatment	ARID1A VAF (%) progression	BAP1 AA-change	BAP1 VAF (%) baseline	BAP1 VAF (%) treatment	BAP1 VAF (%) progression	PBRM1 AA-change	PBRM1 VAF (%) baseline	PBRM1 VAF (%) treatment	PBRM1 VAF (%) progression	PIK3CA AA-change	PIK3CA VAF (%) baseline	PIK3CA VAF (%) treatment	PIK3CA VAF (%) progression	SMAD4 AA-change	SMAD4 VAF (%) baseline	SMAD4 VAF (%) treatment	SMAD4 VAF (%) progression	FBXW7 AA-change	FBXW7 VAF (%) baseline	FBXW7 VAF (%) treatment	FBXW7 VAF (%) progression
1	1st	p.M114V	11.18	-	-	-	-	-	-	-	-	-	-	-	-	-	-	-	-	-	-	-	-	-	-	-	-	-	-	-	-	-	-
1	2nd	p.M114V	7.48			-	-			-	-			-	-			-	-			-	-			-	-			-	-		
2	1st	-	-			-	-			-	-			-	-			-	-			-	-			-	-			-	-		
3	1st	p.L198R	4.58	-	-	-	-	-	-	-	-	-	-	-	-	-	-	-	-	-	-	-	-	-	-	-	-	-	-	-	-	-	-
4	1st	-	-	-	-	-	-	-	-	-	-	-	-	-	-	-	-	-	-	-	-	-	-	-	-	-	-	-	-	-	-	-	-
4	2nd	-	-			-	-			-	-			-	-			-	-			-	-			-	-			-	-		
4	3rd	-	-			-	-			-	-			-	-			-	-			-	-			-	-			-	-		
5	1st	-	-	-	-	-	-	-	-	-	-	-	-	-	-	-	-	p.S39fs	8.07	0.65	-	-	-	-	-	-	-	-	-	-	-	-	-
6	1st	-	-			-	-			-	-			-	-			-	-			-	-			-	-			-	-		
7	1st	-	-			-	-			-	-			-	-			-	-			-	-			-	-			-	-		
8	1st	-	-			-	-			-	-			p.P124R	9.66			-	-			-	-			-	-			-	-		
9	1st	R174X; p.R43	2.64; 6.11	3.94; 2.02	2.45; 3.68	-	-	-	-	-	-	-	-	-	-	-	-	-	-	-	-	-	-	-	-	-	-	-	-	p.R571W	2.93	3.34	4.09
10	1st	-	-			-	-			-	-			-	-			-	-			-	-			-	-			-	-		
11	1st	p.R43H	0.87	-	-	-	-	-	-	-	-	-	-	-	-	-	-	-	-	-	-	p.R38H	2.72	6.92	1.1	-	-	-	-	-	-	-	-
12	1st	-	-			-	-			-	-			-	-			-	-			-	-			-	-			-	-		
13	1st	p.G113D	6.17	4.63	1.47	-	-	-	-	-	-	-	-	-	-	-	-	-	-	-	-	-	-	-	-	-	-	-	-	-	-	-	-
14	1st	-	-			-	-			-	-			-	-			-	-			-	-			-	-			-	-		
15	1st	-	-			-	-			-	-			-	-			-	-			-	-			-	-			-	-		
16	1st	-	-	-	-	-	-	-	-	-	-	-	-	-	-	-	-	-	-	-	-	-	-	-	-	-	-	-	-	-	-	-	-
17	1st	-	-			p.G12V	30.27			p.K974fs	19.37			-	-			-	-			-	-			-	-			-	-		
18	2nd	-	-			-	-			-	-			-	-			-	-			-	-			-	-			-	-		
19	1st	-	-			-	-			-	-			-	-			-	-			-	-			-	-			-	-		
20	1st	-	-			-	-			-	-			-	-			-	-			-	-			-	-			-	-		
21	1st	p.R148K	1.13		4.64	-	-		-	-	-		-	-	-		-	-	-		-	-	-		-	-	-		-	-	-		-
22	1st	-	-	-	-	p.G12V	0.3			-	-			-	-			-	-			p.K111N	0.2			p.R361H	0.2			-	-		
23	1st	-	-	-	-	-	-	-	-	-	-	-	-	c.1730-2A>G	1.52	2.27	1.4	-	-	-	-	-	-	-	-	-	-	-	-	-	-	-	-
24	1st	p.M114V	7.07	1.49	4.46	-	-	-	-	-	-	-	-	-	-	-	-	-	-	-	-	-	-	-	-	-	-	-	-	-	-	-	-
24	2nd	p.M114V	16.73			-	-			-	-			-	-			-	-			-	-			-	-			-	-		

Liquid biopsy (circulating tumor DNA), continued

ID	therapy line	IDH1 AA-change	IDH1 VAF (%) baseline	IDH1 VAF (%) treatment	IDH1 VAF (%) progression	BCL2 AA-change	BCL2 VAF (%) baseline	BCL2 VAF (%) treatment	BCL2 VAF (%) progression	BRAF AA-change	BRAF VAF (%) baseline	BRAF VAF (%) treatment	BRAF VAF (%) progression	CDKN2A AA-change	CDKN2A VAF (%) baseline	CDKN2A VAF (%) treatment	CDKN2A VAF (%) progression	ERBB2 AA-change	ERBB2 VAF (%) baseline	ERBB2 VAF (%) treatment	ERBB2 VAF (%) progression	IDH2 AA-change	IDH2 VAF (%) baseline	IDH2 VAF (%) treatment	IDH2 VAF (%) progression	NRAS AA-change	NRAS VAF (%) baseline	NRAS VAF (%) treatment	NRAS VAF (%) progression
1	1st	-	-	-	-	-	-	-	-	-	-	-	-	-	-	-	-	-	-	-	-	-	-	-	-	-	-	-	-
1	2nd	-	-			-	-			-	-			-	-			-	-			-	-			-	-		
2	1st	-	-			-	-			-	-			-	-			-	-			-	-			-	-		
3	1st	-	-	-	-	-	-	-	-	-	-	-	-	-	-	-	-	-	-	-	-	-	-	-	-	-	-	-	-
4	1st	-	-	-	-	-	-	-	-	-	-	-	-	-	-	-	-	-	-	-	-	-	-	-	-	-	-	-	-
4	2nd	-	-			-	-			-	-			-	-			-	-			-	-			-	-		
4	3rd	-	-			-	-			-	-			-	-			-	-			-	-			-	-		
5	1st	-	-	-	-	-	-	-	-	-	-	-	-	-	-	-	-	-	-	-	-	-	-	-	-	-	-	-	-
6	1st	-	-			-	-			-	-			-	-			-	-			-	-			-	-		
7	1st	-	-			-	-			-	-			-	-			-	-			-	-			-	-		
8	1st	-	-			-	-			-	-			-	-			-	-			-	-			-	-		
9	1st	-	-	-	-	-	-	-	-	-	-	-	-	-	-	-	-	-	-	-	-	-	-	-	-	-	-	-	-
10	1st	-	-			-	-			-	-			-	-			-	-			-	-			-	-		
11	1st	-	-	-	-	-	-	-	-	-	-	-	-	-	-	-	-	-	-	-	-	-	-	-	-	-	-	-	-
12	1st	-	-			-	-			-	-			-	-			-	-			-	-			-	-		
13	1st	-	-	-	-	-	-	-	-	-	-	-	-	-	-	-	-	-	-	-	-	-	-	-	-	-	-	-	-
14	1st	-	-			-	-			-	-			-	-			-	-			-	-			-	-		
15	1st	p.R132C	28.53			-	-			-	-			-	-			-	-			-	-			-	-		
16	1st	-	-	-	-	-	-	-	-	-	-	-	-	-	-	-	-	-	-	-	-	-	-	-	-	-	-	-	-
17	1st	-	-			-	-			-	-			-	-			-	-			-	-			-	-		
18	2nd	-	-			-	-			-	-			-	-			-	-			-	-			-	-		
19	1st	-	-			-	-			-	-			-	-			-	-			-	-			-	-		
20	1st	-	-			-	-			-	-			-	-			-	-			-	-			-	-		
21	1st	-	-		-	-	-		-	-	-		-	-	-		-	-	-		-	-	-		-	-	-		-
22	1st	-	-			-	-			-	-			-	-			-	-			-	-			-	-		
23	1st	-	-	-	-	-	-	-	-	-	-	-	-	-	-	-	-	-	-	-	-	-	-	-	-	-	-	-	-
24	1st	-	-	-	-	-	-	-	-	-	-	-	-	-	-	-	-	-	-	-	-	-	-	-	-	-	-	-	-
24	2nd	-	-			-	-			-	-			-	-			-	-			-	-			-	-		

Supplementary Table 3. Baseline laboratory findings of patients in the study

Baseline laboratory findings	Median Value EHCC (min-max)	Median Value IHCC (min-max)	Unit
Hemoglobin	12.8 (11.6-15.8)	12.2 (9.7-16.1)	g/dl
Platelets	282 (161-511)	240 (173-335)	Giga/l
Leucocytes	7 (6-9.7)	6.7 (4-21.6)	Giga/l
Bilirubin	17 (2-54)	7 (5-171)	µmol/l
Aspartate Aminotransferase (AST)	53 (19-266)	45 (14-329)	U/l
Alanine Aminotransferase (ALT)	56 (12-378)	29 (17-292)	U/l
Gamma-Glutamyl Transferase (GGT)	371 (18-1525)	122 (22-1103)	U/l
Alkaline Phosphatase (AP)	226 (72-682)	133 (39-268)	U/l
Lactate Dehydrogenase (LDH)	238 (170-501)	213 (149-1114)	U/l
CA19-9	114 (5.4-1415)	103 (1.2-10000)	IU/ml
Carcinoembryonic antigen (CEA)	4 (1.2-451)	2.7 (1.3-31)	µg/l
Alpha-fetoprotein (AFP)	6.5 (1-105)	5 (2-41)	µg/l

Supplementary Table 4. Patient risk factors for cholangiocarcinoma in the study cohort

Characteristics	N (%)
**Positive risk factors**	**16/24 (66.7)**
Choledocholithiasis / Cholecystolithiasis	3 (12.5)
Cholecystectomy	2 (8.3)
Hepatitis C	1 (4.2)
Smoker	5 (20.8)
Chronic alcohol abuse	1 (4.2)
Type 2 diabetes	4 (16.7)
**Negative risk factors**	**8/24 (33.3)**

.
